# Human Monoclonal Antibody HCV1 Effectively Prevents and Treats HCV Infection in Chimpanzees

**DOI:** 10.1371/journal.ppat.1002895

**Published:** 2012-08-30

**Authors:** Trevor J. Morin, Teresa J. Broering, Brett A. Leav, Barbra M. Blair, Kirk J. Rowley, Elisabeth N. Boucher, Yang Wang, Peter S. Cheslock, Michael Knauber, David B. Olsen, Steve W. Ludmerer, Gyongyi Szabo, Robert W. Finberg, Robert H. Purcell, Robert E. Lanford, Donna M. Ambrosino, Deborah C. Molrine, Gregory J. Babcock

**Affiliations:** 1 MassBiologics, University of Massachusetts Medical School, Boston, Massachusetts, United States of America; 2 Merck Research Laboratories, West Point, Pennsylvania, United States of America; 3 Department of Medicine, University of Massachusetts Medical School, Worcester, Massachusetts, United States of America; 4 National Institutes of Health, Bethesda, Maryland, United States of America; 5 Department of Virology and Immunology, Texas Biomedical Research Institute and Southwest National Primate Research Center, San Antonio, Texas, United States of America; University of Southern California, United States of America

## Abstract

Hepatitis C virus (HCV) infection is a leading cause of liver transplantation and there is an urgent need to develop therapies to reduce rates of HCV infection of transplanted livers. Approved therapeutics for HCV are poorly tolerated and are of limited efficacy in this patient population. Human monoclonal antibody HCV1 recognizes a highly-conserved linear epitope of the HCV E2 envelope glycoprotein (amino acids 412–423) and neutralizes a broad range of HCV genotypes. In a chimpanzee model, a single dose of 250 mg/kg HCV1 delivered 30 minutes prior to infusion with genotype 1a H77 HCV provided complete protection from HCV infection, whereas a dose of 50 mg/kg HCV1 did not protect. In addition, an acutely-infected chimpanzee given 250 mg/kg HCV1 42 days following exposure to virus had a rapid reduction in viral load to below the limit of detection before rebounding 14 days later. The emergent virus displayed an E2 mutation (N415K/D) conferring resistance to HCV1 neutralization. Finally, three chronically HCV-infected chimpanzees were treated with a single dose of 40 mg/kg HCV1 and viral load was reduced to below the limit of detection for 21 days in one chimpanzee with rebounding virus displaying a resistance mutation (N417S). The other two chimpanzees had 0.5–1.0 log_10_ reductions in viral load without evidence of viral resistance to HCV1. *In vitro* testing using HCV pseudovirus (HCVpp) demonstrated that the sera from the poorly-responding chimpanzees inhibited the ability of HCV1 to neutralize HCVpp. Measurement of antibody responses in the chronically-infected chimpanzees implicated endogenous antibody to E2 and interference with HCV1 neutralization although other factors may also be responsible. These data suggest that human monoclonal antibody HCV1 may be an effective therapeutic for the prevention of graft infection in HCV-infected patients undergoing liver transplantation.

## Author Summary

The majority of individuals infected with hepatitis C virus (HCV) become chronically infected and many go on to develop liver failure requiring liver transplantation. Unfortunately, the transplanted liver becomes infected with HCV in nearly 100% of transplant patients. Current treatments for HCV are poorly tolerated after liver transplantation and graft health is compromised by infection. We have developed a monoclonal antibody called HCV1 that blocks HCV from infecting liver cells in culture. Using chimpanzees as a model for HCV infection, we demonstrate that HCV1 has the ability to prevent HCV infection. We also show that HCV1 can treat chimpanzees chronically infected with HCV and reduce plasma viral load to below the level of detection for a period of 7 to 21 days. The virus that reemerges in the treated chimpanzees was resistant to HCV1 neutralization demonstrating target engagement. Given the ability of HCV1 to protect chimpanzees from HCV infection, we speculate that HCV1 may be beneficial in HCV- infected patients undergoing liver transplant.

## Introduction

More than 180 million people worldwide are infected with hepatitis C virus (HCV) [1], [2] with over 80% developing chronic disease marked by progressive hepatitis, fibrosis and cirrhosis that often results in liver failure [3], [4], requiring transplantation. Unfortunately nearly all donor allografts transplanted into HCV-positive patients become infected with HCV in the early post-transplant period. Standard treatment with interferon–alpha (IFN–α) and ribavirin is poorly tolerated and of limited efficacy in liver transplant recipients [5]–[8] and the recently licensed protease inhibitors have not been extensively studied in this population.

Many lines of evidence suggest broadly-neutralizing antibody preparations may protect from infection with HCV. Before the identification of HCV as the primary cause of non-A, non-B hepatitis, several randomized trials demonstrated that immune serum globulin (ISG) prevented non-A, non-B hepatitis following blood transfusion or sexual exposure [9]–[11]. More recent studies suggest that an early neutralizing antibody response may assist in controlling HCV in the acute phase of infection [12]; however, polyclonal (Civacir) [13] and monoclonal antibody (HCV-Ab^XTL^68) [14] have been tested for efficacy in preventing allograft infection in humans without success possibly due to insufficient dose or neutralizing potency. Furthermore, hepatitis B immune globulin (HBIG) and cytomegalovirus (CMV) immune globulin (Cytogam) have both been used successfully to prevent hepatitis B virus (HBV) and CMV infection, respectively, after liver transplantation [15], [16]. In both instances, combination of antibody plus small molecule anti-viral treatment has been shown to be most effective. Lastly, receipt of HBIG preparations containing anti-HCV antibody was correlated with reduced risk of HCV recurrence in patients undergoing liver transplantation [17]. There is clinical precedence, therefore, for the use of antibody-based therapies to prevent recurrence of viral hepatitis after liver transplantation.

HCV is a member of the *Flaviviridae* family and contains a 9.6 kb positive-stranded RNA genome which encodes a single polypeptide that is cleaved post-translationally into at least ten different proteins. The major HCV surface glycoproteins, E1 and E2, form a non-covalent heterodimer that mediates viral entry into target hepatocytes [18]. Defective lentivirus pseudotyped with E1/E2 envelope glycoproteins (HCVpp) has been shown to infect hepatocytes [19], [20] and HCVpp has become a standard model for studying HCV entry inhibitors. Numerous cellular co-receptors including CD81 [21], claudin-1 [22], occludin [23], scavenger receptor class B type I [24] and others [25] have been identified and shown to play essential roles in the interaction of HCV envelope glycoproteins with hepatocytes. The E2 glycoprotein has been shown to directly interact with cellular receptors [25] and provides an attractive target for monoclonal antibody neutralization.

HCV1 is a human monoclonal antibody isolated using HuMAb mouse (Medarex, Inc., a wholly owned subsidiary of Bristol-Myers Squibb) technology by immunizing with soluble E2 envelope glycoprotein consisting of amino acids 384–660 of E2 [26]. HCV1 recognizes amino acids 412–423 of the HCV E2 envelope glycoprotein (E2 epitope I), a conserved linear epitope in the N-terminus of E2. HCV1 neutralizes a broad range of HCV genotypes (1a, 1b, 2b, 3a and 4a) using HCVpp [26] as well as cell culture-infectious HCV (HCVcc) JFH1/J6 genotype 2a [27]. Alanine scanning mutagenesis has identified positions 413 and 420 as critical for HCV1 binding. These residues are essentially invariable in the Los Alamos HCV sequence database suggesting that changes at these positions are detrimental to the virus. Of interest, antibody response to E2 amino acids 412–423 in chronically-infected HCV patients has been shown to be quite low or nonexistent [28] and nearly all E2 antibodies isolated from infected humans have been directed against conformational epitopes.

Chimpanzees are the only animal other than humans that are permissive to HCV infection and remain the only natural experimental model of HCV infection although expense and ethical concerns limit their use. The chimpanzee provides a model for prevention of initial infection with HCV in the presence of a fully competent immune system that closely models that of humans, an important criteria for the evaluation of a human monoclonal antibody. Also, the levels of HCV replication in chimpanzee are significantly high enough to allow meaningful evaluation of entry inhibitors such as monoclonal antibodies. The infectious inoculum for transmitting HCV to chimpanzees has been carefully characterized [29]. Typically, animals develop viremia shortly after exposure to HCV and can develop long-term infection just as seen in humans. However, the rate at which exposed chimpanzees develop chronic infection is lower than that seen for humans. Chimpanzees have been used to demonstrate that polyclonal antibody has the capacity to prevent initial HCV infection; however, the antibody preparation had to be premixed with the viral inoculum to have a protective effect [30]–[32]. When immunoglobulin containing HCV antibodies were given to chimpanzees shortly after infection as post-exposure prophylaxis, clinical disease was delayed [30]. To our knowledge, no antibody (monoclonal or polyclonal) has ever protected a chimpanzee from initial infection when administered to the chimpanzee prior to viral inoculation. On the whole, chimpanzees are the most appropriate model for the study of HCV-directed human monoclonal antibody therapeutics and may also provide valuable toxicology data that can assess acceptability for human studies.

To determine the potential of HCV1 as a therapeutic for the prevention of HCV infection, chimpanzees were infused with either 50 mg/kg or 250 mg/kg HCV1 followed 30 minutes later with exposure to HCV H77 genotype 1a. HCV1, given at 250 mg/kg, prevented HCV infection of a chimpanzee. Also, HCV1 reduced the viral load of both an acutely-infected and long-term chronically-infected chimpanzee to below the limit of detection for 7 to 21 days followed by a rebound in viral titer. Rebounding virus was shown to harbor mutations in the 412–423 epitope that conferred resistance to HCV1 neutralization by preventing HCV1 binding to the E2 glycoprotein. Interestingly, two chronically-infected chimpanzees only modestly responded to HCV1 treatment and preliminary experiments suggested that components in the chimpanzee sera inhibited the effectiveness of HCV1 neutralization.

## Results

### HCV1 blocks E2 interaction with CD81

**Figure 1:**
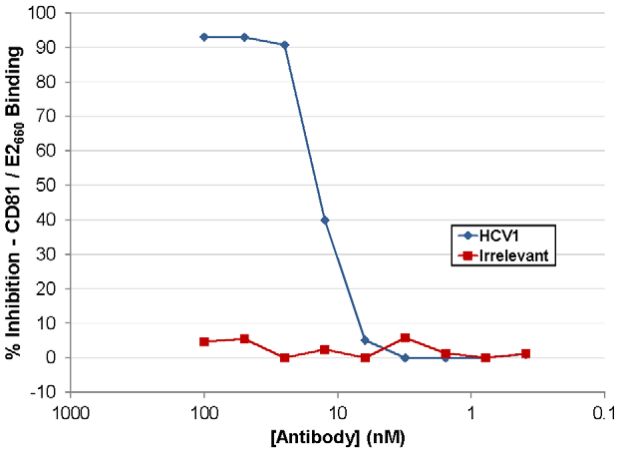
HuMAb HCV1 blocks E2 binding to CD81 LEL. Varying concentration of HCV1 (blue diamonds) or irrelevant human antibody (red squares) were incubated with a constant concentration of E2_660_ and subsequently applied to CD81 LEL-coated to ELISA plates. Binding of E2_660_ to CD81 LEL was detected using an anti-(His)_6_ antibody and the percent inhibition as compared to no antibody plotted.

HCV1 neutralizes HCVpp pseudotyped with E1/E2 derived from a diverse group of genotypes but the mechanism of neutralization had not been determined [26]. Numerous antibodies have been shown to block interaction of E2 with CD81 and this effect correlated with HCV neutralization. The large extracellular loop (LEL) of CD81 was expressed in *E. coli*, purified and coated on ELISA plates. A soluble version of E2 comprising amino acids 384–660 (E2_660_) containing a (His)_6_ epitope tag was produced in CHO cells, purified and quantitated. E2_660_ was incubated with varying concentrations of antibody and the mixture was applied to the CD81 LEL-coated ELISA plates. Binding of E2_660_ to CD81 LEL was detected using an anti-(His)_6_ antibody and the results, graphed as percent inhibition of the CD81 LEL/E2_660_ interaction, are shown in **Figure 1**. HCV1 was able to inhibit E2_660_ interaction with CD81 LEL (>90% at the highest antibody concentrations tested) whereas an irrelevant human antibody had no impact on binding. These results demonstrate that HCV1 likely neutralizes HCV by blocking E2 interaction with the CD81 receptor found on target cells.

### HuMAb HCV1 protects a chimpanzee from initial challenge with HCV

**Figure 2:**
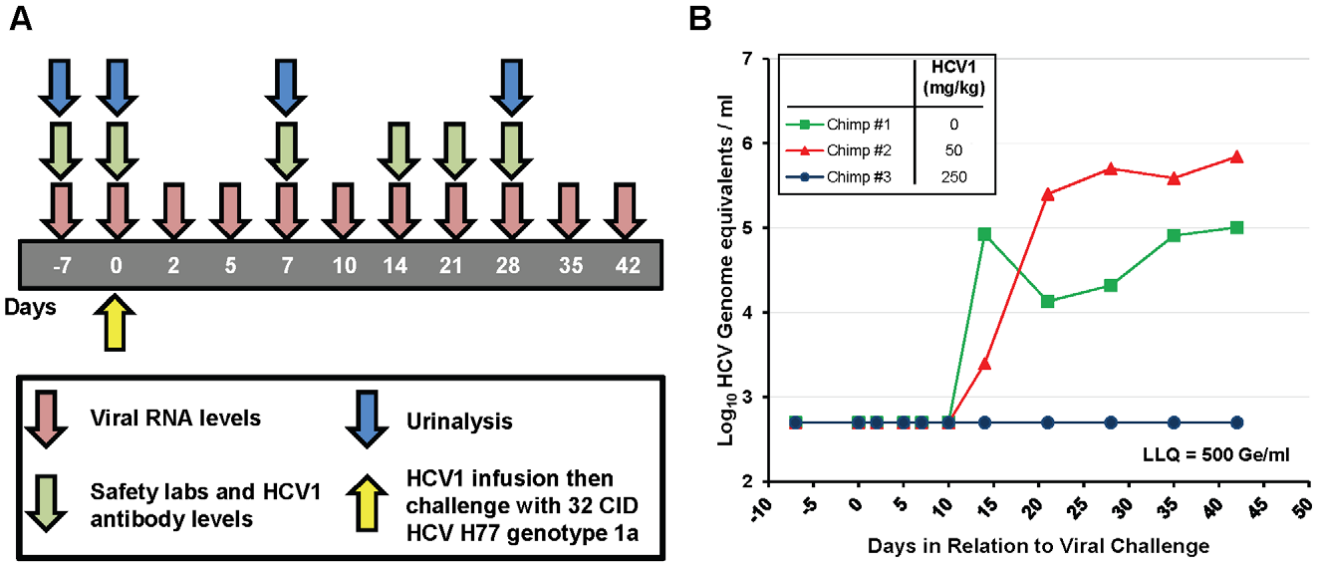
HuMAb HCV1 protects chimpanzees from initial infection with HCV. **A**. A schematic representation of the chimpanzee protection study is shown. The first 42 days of the experiment are represented by the gray bar with the days of sampling listed. Viral RNA levels (red), safety labs and HCV1 antibody levels (green) and urinalysis (blue) were measured at the time points listed below the arrows. H77 genotype 1a HCV viral challenge (32 chimpanzee infectious doses (CID)) was administered 30 minutes following infusion with HCV1 antibody (day 0, yellow). **B**. Serum viral RNA levels were measured from day −7 to day +42 post-infection using quantitative PCR and were reported as genome equivalents (Ge)/ml. The legend denotes the dose of HCV1 (mg/kg) each chimpanzee received prior to virus infusion. The lower limit of quantification for the qPCR was 500 Ge/ml.

HCV1 was chosen as the lead candidate antibody for use in chimpanzee studies. Three HCV-naïve, healthy chimpanzees were challenged with 32 chimp infectious doses (CID) of HCV genotype 1a strain H77. Thirty minutes prior to infusion with the HCV challenge, chimpanzee #2 received a single intravenous infusion of 50 mg/kg HCV1 and chimpanzee #3 received a single intravenous infusion of 250 mg/kg HCV1. Chimpanzee #1 did not receive HCV1 and served as the untreated control. It is important to note that the viral inoculum was not pre-incubated with HCV1 antibody *ex vivo*. **Figure 2A** is a schematic representation of the sampling and testing performed over the first 42 days of the 20 week study. HCV RNA in the chimpanzee sera was measured using RT-PCR with a lower limit of quantification (LLQ) equal to 500 genome equivalents (Ge)/ml. Infusion with HCV1 at both 50 mg/kg and 250 mg/kg was well tolerated and no infusion-related reactions were observed. There were no significant changes in hematology, serum chemistries or urinalysis following HuMAb infusion (data not shown). Viral load measurements demonstrated that chimpanzee #1 (untreated control) and chimpanzee #2 (50 mg/kg HCV1) were infected with HCV by 14 days after challenge (**Figure 2B**). HCV RNA was not detected in chimpanzee #3 (250 mg/kg) during the initial 42 days shown in **Figure 2B** and remained below the limit of detection for the duration of the study (140 days) at which time the experiment was terminated. The serum concentration of HCV1 was determined at multiple time points in chimpanzee #3 using an ELISA specific for E2 amino acids 412–423 and the terminal half-life of HCV1 was estimated to be 11.7 days. These results demonstrate that HCV1 was able to prevent initial HCV infection in one chimpanzee dosed with 250 mg/kg but was not protective in another chimpanzee given 50 mg/kg.

To determine if the treatment failure of the chimpanzee that received 50 mg/kg HCV1 was due to mutation in the viral envelope glycoprotein that provided resistance to HCV1, we employed RT-PCR to amplify and sequence the entire E1/E2 envelope glycoprotein coding region from HCV RNA in the serum of untreated chimpanzee #1 at 21 days after viral challenge and from the 50 mg/kg-treated chimpanzee #2 at 35 days after viral challenge. Different days were chosen for viral sequencing based on the availability of serum samples. A minimum of 8 distinct viral clones from each infected chimpanzee were sequenced and compiled. The derived sequences were translated and the amino acid sequences were compared to the consensus amino acid sequence present in the viral inoculum (H77 genotype 1a). For each chimpanzee #1 and #2, only three amino acid positions were altered from that of the HCV H77 genotype 1a virus (**Table 1**). Both the untreated chimpanzee #1 and the 50 mg/kg-treated chimpanzee #2 had no alterations in amino acids 412–423 when compared to the consensus H77 sequence. Untreated chimpanzee #1 had 3 positions, 394, 434 and 444, that were divergent from the H77 consensus sequence. Treated chimpanzee #2 also had three amino acid positions that did not match those found in the H77 consensus sequence and was different than those in chimpanzee #1, residues 391, 401 and 608. Based on the epitope of HCV1, none of the sequence alterations found in either chimpanzee #1 or chimpanzee #2 were predictive of HCV1-resistant virus.

**Table 1:** Amino acid alterations identified in H77 HCV E1/E2 envelope glycoproteins after initial viral infection in untreated (#1) and treated (#2) chimpanzees.

	Amino Acid Position																	
	391	394	401	412	413	414	415	416	417	418	419	420	421	422	423	434	444	608
H77	S	R	G	Q	L	I	N	T	N	G	S	W	H	I	N	E	Q	M
Chimpanzee #1 (day +21)	-	H	-	-	-	-	-	-	-	-	-	-	-	-	-	D	R/Y	-
Chimpanzee #2 (day +35)	N	-	S	-	-	-	-	-	-	-	-	-	-	-	-	-	-	V

To determine if the lack of virologic control in chimpanzee #2 correlated with resistance to neutralization, lentiviral pseudovirus was generated. This was accomplished by co-transfection of HEK-293T/17 cells with the E1/E2 sequences from the untreated chimpanzee #1 or the 50 mg/kg-treated chimpanzee #2 and the lentiviral backbone with a defective native glycoprotein gene and an engineered luciferase reporter gene (HCVpp). HCVpp was harvested from the culture supernatants, concentrated and stored frozen in aliquots. Varying dilutions of HCV1 were incubated with pseudovirus from chimpanzee #1 (day +21) and chimpanzee #2 (day +35), CHP1+21–HCVpp and CHP2+35–HCVpp, respectively, and HCV1 neutralization capacity assessed. HCV1 was able to neutralize both CHP1+21–HCVpp and CHP2+35–HCVpp equivalently to H77–HCVpp (data not shown). These data demonstrate that the inability of HCV1 to protect chimpanzee #2 from infection was not due to HCV1-resistant virus.

### HCV1 neutralizes HCV in an acutely-infected chimpanzee

**Figure 3:**
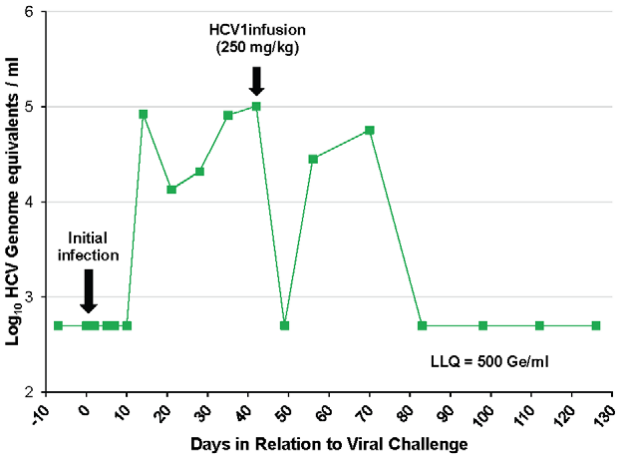
HCV1 reduces viral load to below the limit of quantification in an acutely-infected chimpanzee. Chimpanzee #1 was administered 32 CID H77 genotype 1a HCV on day 0 and circulating virus was detected at 14 days after initial challenge. Forty-two days following viral challenge, an infusion of 250 mg/kg HCV1 was delivered and circulating viral titer measured by qPCR at days −7 to +126 is shown (LLQ = 500 Ge/ml).

To determine if HCV1 has the capacity to treat an acutely-infected animal, the untreated control chimpanzee #1 was administered 250 mg/kg HCV1 42 days following initial infection with 32 CID H77 virus. Viral load was measured at day +49 and HCV could not be detected in the serum (**Figure 3**). At day +56, two weeks following HCV1 administration, viral load was found to have rebounded and continued to persist through day +72, at which time the chimpanzee naturally cleared the virus (**Figure 3**). These results suggest that HCV1 provided strong neutralizing activity in the setting of recent infection and it is worth noting that HCV1 antibody levels in the serum were still high at the time of viral rebound (data not shown).

Given the complete suppression of circulating HCV followed by rapid rebound of the virus in this chimpanzee we assessed whether rebounding virus had developed resistance mutations that would allow for viral escape from HCV1 neutralization. RT-PCR was performed on serum samples from chimpanzee #1 on day +42 before HCV1 treatment and day +56 (two weeks following treatment) to determine sequence changes in the viral envelope glycoprotein gene following receipt of HCV1 antibody. The entire E1/E2 coding region was amplified and multiple clones were sequenced. The nucleotide sequence was translated and it was determined that nearly all amino acids were identical between the day +42 and day +56 sequences with the exception of position 415. In all of the viral sequences identified at day +56, N415 had been altered to either N415D or N415K, in equal distribution (10 of 20 sequenced clones for each mutation) (**Table 2**). The N415K mutation had previously been identified *in vitro* and H77–HCVpp bearing N415K E1/E2 had been shown to be resistant to HCV1 neutralization [26]. Four E2 residues, 480, 612, 615 and 618, showed minor alterations in that the amino acid usage was mixed at day +56 as compared to that seen at day +42 (**Table 2**).

**Table 2:** Amino acid alterations in E1/E2 envelope glycoproteins following viral rebound in treated chimpanzee #1.

	Amino Acid Position															
	412	413	414	415	416	417	418	419	420	421	422	423	480	612	615	618
Pre-HCV1 (day +42)	Q	L	I	N	T	N	G	S	W	H	I	N	L/P	P	L	Y
Post-rebound (day +56)	-	-	-	**K/D**	-	-	-	-	-	-	-	-	L/H	P/L	L/F	Y/F

To determine if the N415K/D changes resulted in resistance to HCV1, HCVpp were generated using E1/E2 sequences from chimpanzee #1 identified on both day +42 and day +56. As expected HCVpp bearing envelope glycoproteins cloned from day +42 virus (CHP1+42–HCVpp) were neutralized by HCV1 equivalently to H77–HCVpp (**Table 3**). In contrast, HCVpp incorporating E1/E2 glycoproteins from day +56 virus (CHP1+56–HCVpp) were completely resistant to HCV1 neutralization even at the highest concentration of antibody tested (1000 nM). To confirm that resistance was a result of the N415K/D mutation in CHP1+56 E1/E2 envelope glycoprotein, site-directed mutagenesis was performed to revert the 415 position back to asparagine. As expected, this mutation restored sensitivity to HCV1 neutralization (**Table 3**). In addition, site-directed mutagenesis was performed to incorporate the N415K or N415D mutation into the E1/E2 gene from the H77 isolate. Both mutations at position 415 resulted in H77–HCVpp that were completely resistant to HCV1 neutralization. These results confirm that mutation at position 415 of the E1/E2 envelope glycoprotein following treatment with HCV1 allowed the virus present in chimpanzee #1 to escape neutralization. Of note, H77–HCVpp containing engineered E1/E2 alterations L480H, P612L, L615F or Y618F (found in virus present at day +56) were all neutralized by HCV1 equivalently to wild–type H77–HCVpp (data not shown).

**Table 3:** IC_50_ values of HCV1 neutralization of various chimpanzee-derived HCVpp.

Source of E1/E2	Day to HCV1	E1/E2 mutation	IC_50_ (nM)
Chimpanzee #1	+42	-	3.6
Chimpanzee #1	+56	415K#	>1000
Chimpanzee #1	+56	415D#	>1000
Chimpanzee #1	+56	K415N	4.3
Chimpanzee #1	+56	D415N	3.9
H77	-	-	5.4
H77	-	N415K	>1000
H77	-	N415D	>1000
Chimpanzee A	−8	-	2.1
Chimpanzee B	−8	-	1.6
Chimpanzee C	−8	-	0.53
Chimpanzee B	+35	417S#	>1000
H77	-	N417S	*Non-infectious*
H77	-	N417S/Q444R	>1000
H77	-	Q444R	2.9

# Mutation induced by treatment with HCV1, not site-directed mutagenesis.

### HCV1 treatment of chronically-infected chimpanzees

**Figure 4:**
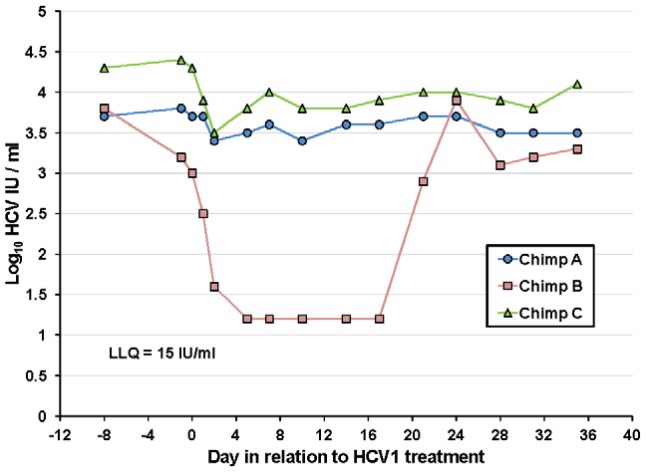
Treatment of chronically-infected chimpanzees with HuMAb HCV1. Three chronically-infected chimpanzees, A, B and C, were infused with 40 mg/kg HCV1 at day 0 of the experiment. Circulating viral titer was assessed using qPCR (LLQ = 15 IU/ml) for the duration of the study and the results until day +35 were graphed.

Although HCV1 was able to reduce viral load to below the level of detection in an acutely-infected chimpanzee, this protection was not durable and resulted in the emergence of resistant virus. To determine the efficacy of HCV1 in treating chronically-infected chimpanzees, a single intravenous infusion of HCV1 at 40 mg/kg was administered to three chronically-infected (>5 years) chimpanzees (genotype 1a). This dose was the maximum allowable dose as determined by the IACUC at the institution where this specific study was performed. Each infusion was well tolerated and no adverse reactions were observed. Viral load was measured during the course of the study and the results were plotted through day +35 in **Figure 4**. Two of the chimpanzees, A and C, had minor reductions in viral load immediately following the infusion, 0.5 log_10_ and 1 log_10_, respectively. In contrast, the viral load in chimpanzee B rapidly diminished to below the level of detection by day +5. HCV RNA remained below the level of detection until day +21 and returned to pre-treatment levels by day +24.

To determine if the rebound in viral load in chimpanzee B was a result of the emergence of escape virus resistant to HCV1, the entire E1/E2 gene from chimpanzee B at day +35 was isolated and sequenced and compared to sequences obtained from this same chimpanzee prior to HCV1 treatment (day −8) and the results are shown in **Table 4**. 100% of the sequences identified at day +35 had two significant alterations that were not seen in any day −8 clones, one at position 417 (N to S) and the other at position 444 (Q to R). The mutation N417S is within the epitope for HCV1 (amino acids 412–423) and suggested that this alteration may provide resistance to HCV1 neutralization.

**Table 4:** Amino acid alterations in E1/E2 envelope glycoproteins following viral rebound in treated chimpanzee B.

	Amino Acid Position														
	330	412	413	414	415	416	417	418	419	420	421	422	423	444	610
Pre-HCV1 (day −8)	A	Q	L	I	N	T	N	G	S	W	H	I	N	Q	N
Post-rebound (day +35)	A/G	-	-	-	-	-	**S**	-	-	-	-	-	-	R	N/D

To ascertain if the changes at position 417 and 444 conferred resistance to HCV1 neutralization, HCVpp was generated using E1/E2 sequence isolated from chimpanzee B at day +35 (CHPB+35–HCVpp). Varying concentrations of HCV1 were applied to CHPB+35–HCVpp and neutralization potency determined. HCV1 was unable to neutralize CHPB+35–HCVpp at any concentration tested demonstrating that the virus was completely resistant to HCV1 (**Table 3**). A neutralizing HCV HuMAb (96-2) directed against E2 epitope II (amino acids 432–443) was able to neutralize this virus equivalently to H77–HCVpp (data not shown). Also, mutation of the S417 to N417 in CHPB+35–HCVpp restored sensitivity to HCV1 neutralization (data not shown).

To confirm that the N417S mutation was responsible for escape from HCV1 neutralization, we engineered this mutation into the H77 virus E1/E2 envelope glycoprotein and generated H77_N417S_–HCVpp. Interestingly, this mutation rendered the pseudovirus non-infectious and we were initially unable to assess the impact of this mutation on HCV1 neutralization (**Table 3**). Given that the N417S and Q444R both arose in the chimpanzee upon viral rebound we created H77 pseudovirus bearing both of these mutations (H77_N417S/Q444R_–HCVpp). Addition of the Q444R mutation to the N417S mutation restored H77–HCVpp infectivity suggesting that the Q444R mutation was compensatory to N417S. HCV1 was unable to neutralize H77_N417S/Q444R_–HCVpp at the highest concentration tested (1000 nM, **Table 3**) whereas the epitope II-specific antibody, 96-2, could potently neutralize this HCVpp (data not shown). H77–HCVpp was also generated solely containing the Q444R mutation (H77_Q444R_–HCVpp) and HCV1 neutralized this HCVpp similarly to wild type H77–HCVpp (**Table 3**) suggesting that the mutation at position 444 does not confer resistance to HCV1.

### HCV1 has the capacity to neutralize HCVpp pseudotyped with E1/E2 derived from poorly-responding chimpanzees

Since HCV1 was able to reduce virus to below the limit of detection in chimpanzee B but did not provide robust antiviral activity in either chimpanzee A or C, it was formally possible that HCV1 was unable to neutralize the predominant virus in these two chimpanzees. To test this hypothesis, E1/E2 gene sequences were isolated from serum obtained from each of the three chimpanzees 8 days prior to treatment with HCV1. The dominant sequence from each chimpanzee was cloned into an expression vector and HCVpp were pseudotyped with each synthesized envelope glycoprotein. HCV1 was able to potently neutralize HCVpp harboring envelope glycoprotein from all day −8 chimpanzee sequences (**Table 3**) suggesting that virus in all three chimpanzees was sensitive to HCV1.

We then examined whether the virus found in chimpanzees A and C had developed mutations that were resistant to HCV1 neutralization shortly after treatment. We sequenced isolated clones from each chimpanzee 8 to 10 days following HCV1 infusion. No significant alterations were found in the coding region for E1/E2 following treatment with HCV1, and no changes were found in the 412–423 epitope (data not shown). To demonstrate that there were not changes that conferred resistance, we generated HCVpp using E1/E2 isolated from chimpanzee A and C 10 days following treatment with HCV1. HCV1 had comparable neutralization potency against HCVpp containing E1/E2 isolated either eight days prior or ten days following treatment with HCV1 from both chimpanzees (data not shown). These results suggest that HCV1-resistant virus had not developed in the two chimpanzees with a minor reduction in viral load following HCV1 treatment.

### Serum from poorly-responding chimpanzees inhibits HCV1 neutralization of HCVpp

**Figure 5:**
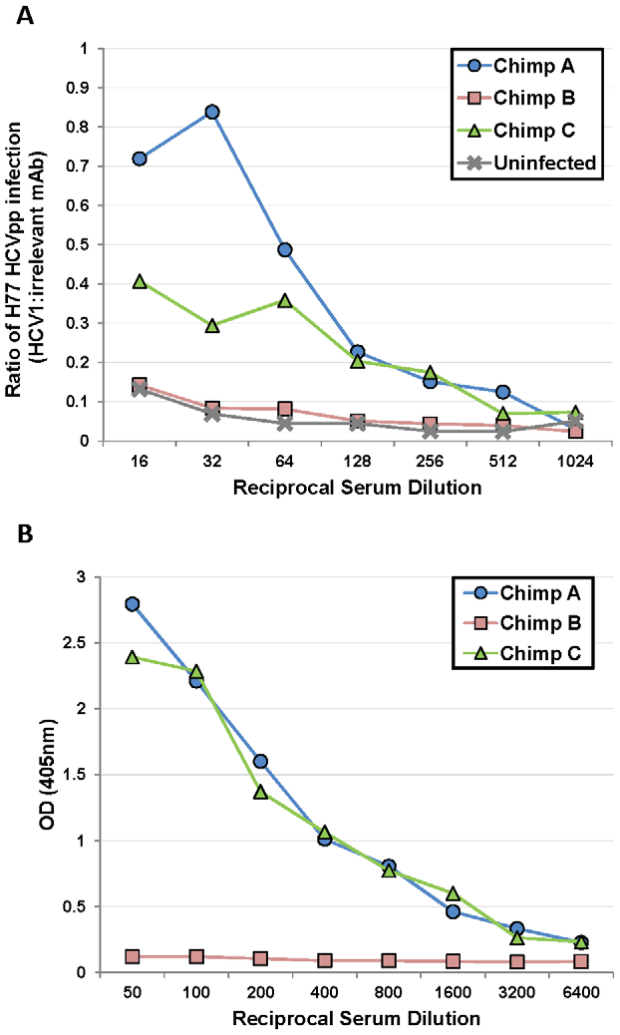
Serum from poorly-responding chimpanzees inhibits HCV1 neutralization of H77–HCVpp. **A**. Serum from the three chronically-infected chimpanzees, collected prior to HCV1 infusion, and serum from an uninfected chimpanzee were mixed at varying dilutions (1∶16 to 1∶1024) with a constant concentration (100 nM) of HCV1 or irrelevant human antibody. The mixture was incubated with H77 genotype 1a HCVpp and applied to Hep3B cells for 72 hours. Bright-Glo luciferase detection reagent was used to quantitate infection of the luciferase containing HCVpp. Light output was quantified and the counts per second (CPS) for the HCV1 sample was divided by the CPS for the irrelevant antibody sample for each dilution of the serum. The CPS quotient as related to each serum dilution was plotted. A value of 1 would represent no neutralization of HCVpp with HCV1 and a value approaching 0 would signify that HCV1 neutralized 100% of the HCVpp. **B**. E2_660_ envelope glycoprotein ELISA was performed on varying dilutions of chronically-infected chimpanzee serum collected prior to HCV1 infusion. Bound antibody was detected employing goat anti-human secondary antibody conjugated to alkaline phosphatase, followed by development with PNPP substrate, and the absorbance at 405 nm for each dilution was plotted.

Because HCV1 neutralized HCVpp from chimpanzees A and C before and after treatment with equivalent potency, we speculated that components of the serum, present in the chimpanzees with low-level response to HCV1 (A and C) but not in serum from the chimpanzee with complete response (B), inhibited HCV1 neutralization. H77–HCVpp was incubated with 100 nM HCV1 or an irrelevant human antibody in the presence of varying concentrations of serum from each of the three chronically HCV-infected chimpanzees as well as serum from an uninfected chimpanzee and infectivity was assessed. Chimpanzee serum alone had a profound and variable impact on H77–HCVpp infection by either enhancing or inhibiting infection depending on the dilution used (data not shown). To overcome the variability introduced by serum in the assay, a ratio of infection in the presence of HCV1 as compared to an irrelevant antibody was determined and plotted (**Figure 5A**). A ratio of 1 indicates that HCV1 was unable to neutralize HCVpp in the presence of chimpanzee serum and a ratio approaching 0 would indicate complete neutralization of HCVpp. When H77–HCVpp was incubated with 100 nM HCV1 (fully neutralizing concentration) in the presence of a 1∶16 serum dilution from chimpanzee A or C, the infection, expressed as a ratio of HCV1 to irrelevant antibody, was 0.72 and 0.4 respectively demonstrating significant HCVpp infection suggesting that HCV1 neutralization was impaired. In contrast, this ratio was only 0.15 for serum from both the uninfected chimpanzee and chimpanzee B, consistent with a low level of HCVpp infection, suggesting that HCV1 was able to neutralize HCVpp effectively. Infection in the absence of chimpanzee serum resulted in a ratio near 0.1 (data not shown) suggesting effective HCV1 neutralization. These data demonstrate that a component in the serum from both chimpanzees A and C interfered with HCV1 neutralization of H77–HCVpp and this factor is not present in the serum of chimpanzee B or an uninfected chimpanzee.

We postulated that non-neutralizing antibody present in the serum of chimpanzees A and C may be responsible for abrogating the neutralizing activity of HCV1. We performed an ELISA to measure the serum concentration of antibody directed against soluble E2 envelope glycoprotein (E2_660_). E2_660_ was coated on ELISA plates and varying dilutions of chimpanzee serum was applied and detected with goat anti-human polyclonal antibody. Plates were developed and the results plotted in **Figure 5B**. Chimpanzees A and C had high serum concentrations of antibody directed against E2_660_ whereas chimpanzee B had very low antibody levels to this protein (**Figure 5B**). These data suggest that endogenous antibody specific to E2 in the serum of chimpanzees A and C may be capable of interfering with neutralization of HCV with the HCV1 antibody and potentially explains the differences in outcomes after treatment of the animals with chronic infection. Alternatively, the findings may be related to the dose of antibody given, and as was seen in the prevention study, a higher dose of antibody could have been effective for HCV neutralization.

### Mutations at E2 amino acids 415 and 417 disrupt HCV1 binding

We previously reported that E2_660_ soluble glycoprotein harboring an N415K mutation produced by CHO cells in defined, serum-free media (CD-CHO, Invitrogen) was bound strongly by HCV1 yet HCV1 could not neutralize H77_N415K_–HCVpp [26]. This result led to speculation that HCV1 binding required amino acid residues distant to amino acids 412–423, although we believed this to be unlikely.

**Figure 6:**
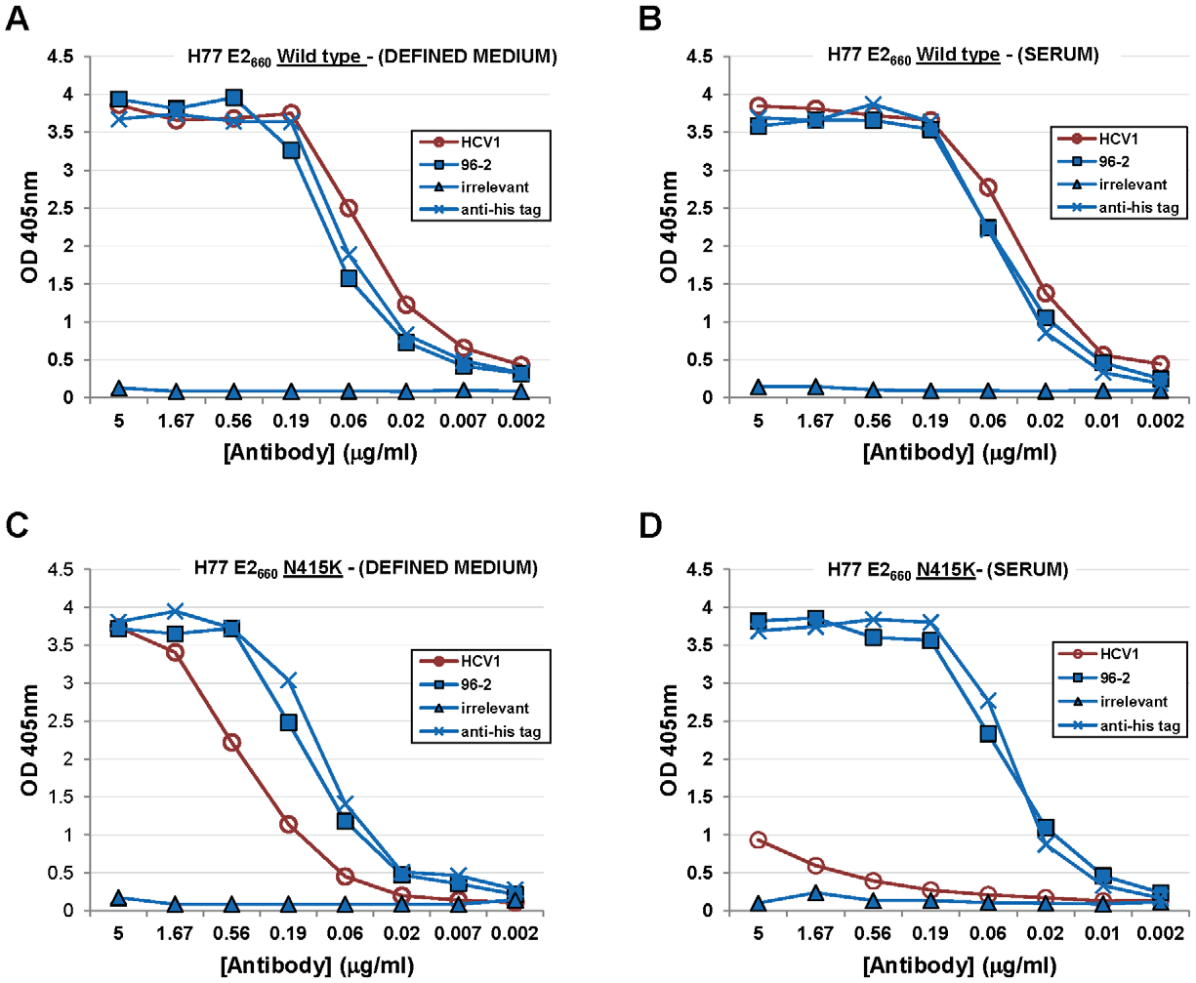
HCV1 does not bind mutant E2 envelope glycoprotein produced in serum-containing medium. A vector encoding wild-type E2_660_ was transfected into CHO cells grown in defined media (**A**) or serum-containing medium (**B**). In addition, a vector encoding E2_660_–N415K was also transfected into CHO cells grown in defined media (**C**) or serum-containing media (**D**). All proteins were purified from culture supernatant using nickel-affinity chromatography, quantified, assessed for purity and coated on ELISA plates. Varying dilutions of HCV1 (red circles) was applied to each protein and binding assessed. In addition, varying dilutions of 96-2 (epitope II specific, blue squares), irrelevant antibody (blue triangles) and a mouse antibody specific to the (His)_6_ tag (blue crosses) at the C-terminus of E2_660_ were assayed to control for protein coating and non-specific binding. For HCV1, 96-2 and irrelevant mAb, bound antibody was detected employing goat anti–human secondary antibody conjugated to alkaline phosphatase, followed by development with PNPP substrate, and the absorbance at 405 nm for each dilution was plotted. For the mouse anti-(His)_6_ antibody, goat anti-mouse secondary antibody was used.

HCV1 binding to E2 harboring mutations at positions 415 and 417 and produced under various cell culture conditions was assessed. HCVpp are typically produced from HEK-293T/17 cells in bovine serum-containing media and we speculated that E2 glycoprotein produced in media containing serum was not equivalent to E2_660_ (H77–derived) produced in defined media. In fact, soluble glycoprotein produced in CD-CHO had significantly different mobility than E2_660_ produced in serum-containing media when analyzed by SDS-PAGE (data not shown). We performed binding studies (ELISA) to determine if HCV1 had differential recognition of E2_660_ depending on the method of protein production. Wild-type E2_660_ and E2_660_ with an N415K mutation (E2_660_–N415K) were produced from CHO cells in either serum-containing media or serum–free CD-CHO media. The four proteins were coated on ELISA plates and HCV1 binding at varying concentrations assessed. HCV1 strongly bound to wild type E2_660_ regardless of the type of media used to grow the E2_660_–producing CHO cells (**Figures 6A and 6B**). HCV1 bound strongly to E2_660_–N415K produced in defined media (**Figure 6C**) though less well than to wild-type E2_660_ produced under the same conditions. However, E2_660_–N415K produced from CHO cells in serum-containing media was bound very weakly by HCV1 showing minimal binding even at 5 µg/ml (**Figure 6D**). Both the anti–(His)_6_ specific antibody and the epitope II-specific 96-2 antibody recognized all proteins equivalently (**Figure 6A–D**). These experiments were repeated using E2_660_–N415D and E2_660_–N417S (data not shown) and we observed the same result as shown for E2_660_–N415K. These data demonstrate that mutation at amino acids 415 or 417 of the E2 envelope glycoprotein abrogate HCV1 binding and thus neutralization capacity.

## Discussion

Chronic infection with HCV leads to liver failure in 5–10% of patients with liver transplant being the only treatment option. In the setting of liver transplantation for chronic hepatitis C nearly all liver allografts become infected with HCV and the long-term survival of the graft is compromised by infection [33]–[39]. The clinical course of recurrent hepatitis C is often aggressive resulting in accelerated allograft cirrhosis and increased risk of graft failure and death [34], [40], [41]. Treatment with pegylated-IFN–α and ribavirin is often attempted, but the limited efficacy is balanced against the significant risk of adverse events in this population [8], [42]. The first FDA-licensed HCV direct-acting antiviral agents, boceprevir and telaprevir, are indicated for chronic HCV infection but their tolerability profile will likely limit their use in patients that have undergone liver transplantation. Novel, safe and efficacious therapies to prevent HCV recurrence are needed, particularly because of the limited supply of organs for transplantation. Treatment during liver transplantation with human monoclonal antibody capable of neutralizing HCV infection of the liver allograft is an attractive potential therapy because of the favorable tolerability profile of human monoclonal antibodies, IV administration, and precedent of HBV neutralization during liver transplant with human polyclonal antibodies. Because HCV does not integrate into the host genome, viral clearance and cure should be attainable in theory. However, past clinical and chimpanzee experiments have given mixed results for the success of HCV specific antibodies for prevention of infection.

The HCV1 human monoclonal antibody can potently neutralize a broad range of HCV genotypes in the HCVpp system and recognizes a conserved linear epitope in the E2 envelope glycoprotein (epitope I, amino acids 412–423) [26]. Neutralization of circulating virus with a monoclonal antibody prior to engraftment may prevent liver infection in the transplant patient population. As a surrogate for graft infection, we used a model of initial infection in chimpanzees. HCV1, at doses of 50 mg/kg or 250 mg/kg, was infused into chimpanzees 30 minutes prior to challenge with 32 CID of H77 genotype 1a virus. The chimpanzee receiving 250 mg/kg was completely protected from HCV infection whereas the chimpanzee receiving 50 mg/kg HCV1 was not protected. It is unclear if the higher dose of HCV1 is required for protection from initial infection in chimpanzees given that only one chimpanzee per cohort was assessed. It is formally possible that HCV1 can only protect a subset of chimpanzees from initial infection regardless of dose and the limited number of chimpanzees tested did not allow for statistical significance. Also, it was formally possible that the chimpanzee that received the 250 mg/kg dose was refractory to HCV infection; however, in subsequent experiments, this chimpanzee was shown to be permissive to HCV genotype 1b infection (data not shown). Sequence analysis of the virus from the chimpanzee that received the lower dose of HCV1 did not reveal any sequence alterations that would be predicted to resist HCV1 neutralization. In fact, the sequences identified in this chimpanzee were nearly an exact match for the H77 consensus sequence and any minor variation was also observed in the untreated chimpanzee. Given the expense and ethical issues associated with chimpanzee studies, true dose-ranging studies of HCV1 to determine a therapeutic level were not performed.

To our knowledge, this is the first demonstration that pre-administered antibody can protect chimpanzees from initial infection with HCV. This study is the first report of an antibody directed against E2 amino acids 412–423 tested in chimpanzees and it is possible that this epitope is the appropriate target of a protective monoclonal antibody. This is supported by the fact that polyclonal antibody, which does not protect from initial infection in this model, has been shown to contain very little antibody directed against the HCV1 epitope. Another possibility is that we delivered a higher dose of antibody than has been delivered previously and this fact led to our success. At this time it is unclear which of these possibilities are correct and experiments directly comparing HCV1 to other neutralizing antibodies would need to be performed.

The anti-viral neutralizing activity of HCV1 was also demonstrated in acutely-infected and chronically-infected chimpanzees. For the initially untreated chimpanzee that developed an acute infection (chimpanzee #1), a dose of 250 mg/kg was able to suppress viral load to an undetectable level before viral rebound was noted 14 days following HCV1 infusion. For the chronically-infected chimpanzees, a single dose of 40 mg/kg was able to suppress viral titer to below the level of detection for 21 days in one chimpanzee with the other two chimpanzees demonstrating a 0.5 and 1.0 log_10_ reduction in viral load within four days of treatment. For the two chimpanzees treated with HCV1 (acutely- and chronically-infected) in which the viral load was suppressed to undetectable levels, escape mutations were detected in the 412–423 E2 epitope upon viral rebound. Specifically, N417S and N415K/D mutations were observed in 100% of the HCV sequences isolated from the chronically-infected and acutely-infected chimpanzee, respectively, indicating strong selective pressure of the antibody on the virus of both responding chimpanzees. Not surprisingly, HCV1 was unable to neutralize N415K/D–HCVpp bearing glycoproteins derived from the acutely-infected chimpanzee or H77–HCVpp engineered to contain these mutations. HCV1 was also unable to neutralize HCVpp pseudotyped with E1/E2–N417S derived from the chronically-infected chimpanzee. Interestingly, N417S mutant H77–HCVpp was not infectious in our culture system. However, the introduction of the Q444R in addition to the N417S mutation in H77–HCVpp restored infectivity and HCV1 was then unable to neutralize this pseudovirus. The N417S mutation identified in chimpanzee B following viral rebound was accompanied by a Q444R mutation which presumably was compensatory to maintain infectivity of the virus bearing this N417S mutation.

It is not clear why two of the chronically-infected chimpanzees had an incomplete virologic response to treatment with HCV1. Virus isolated from these two poorly-responding chimpanzees had E1/E2 glycoprotein sequences nearly identical to the viral sequences prior to HCV1 infusion suggesting that escape virus did not develop in these chimpanzees. Also, HCV1 was able to neutralize HCVpp bearing envelope glycoproteins derived from both poorly-responding chimpanzees with a similar potency to H77–HCVpp indicating that HCV1 had the capacity to neutralize virus from these two chimpanzees. Interestingly, in the treatment model of chronic infection, sera from the two poorly-responding chimpanzees, but not the responding chimpanzee, were able to inhibit HCV1 neutralization of H77–HCVpp. These chimpanzees received a dose of 40 mg/kg HCV1 rather than the 250 mg/kg used in the prevention study due to IACUC restrictions. It is possible that a higher dose of HCV1 may have reduced the viral load in all three chronically-infected chimpanzees rather than one of three by overcoming the neutralization inhibition imposed by the poorly-responding chimpanzee sera. Given previous literature reports, the inhibition could be due to competing non-neutralizing antibodies, serum lipoproteins, or other as yet unidentified factors. Due to the small number of animals and the limited supply of serum from the animals, we were not able to determine the nature of the serum factor that inhibited the neutralization of HCVpp. It is possible that endogenous anti-HCV antibodies directed against epitope II of E2 may interfere with HCV1 potency. It has been suggested that epitope II-specific antibodies in both chimpanzees and humans inhibit epitope I-directed antibodies [43], [44] though these data have recently been challenged [45]. Using ELISA we demonstrated that chronically-infected chimpanzees with an incomplete virologic response to HCV1 did possess high concentrations of anti-E2_660_ antibody in contrast to the low concentrations found in the chimpanzee with complete virologic response. It should be noted that we also performed ELISA on the chimpanzee serum to determine the titers of epitope I- and II-specific antibodies and none of the three chimpanzees had appreciable antibody to these two epitopes (data not shown). If endogenous chimpanzee non-neutralizing antibodies do interfere with HCV1, these antibodies are most likely not directed to epitope II but to other regions of the envelope glycoproteins. We were unable to deplete chimpanzee antibody from the serum and perform HCVpp assays due to the limited supply of chimpanzee serum from this study.

It has also been hypothesized that serum lipoproteins coating HCV virions present in sera may interfere with epitope I-directed antibodies such as HCV1 [46], [47] though we have not been able to reproduce these results in our laboratory (Babcock, unpublished observations). The chimpanzees that did fully respond to HCV1 treatment would have to have differences in lipoprotein/virion coating from the poorly-responding chimpanzees and this appears to be unlikely. To determine the exact cause of the failure of HCV1 to suppress viral load in 2 of 4 chimpanzees (antibody, lipoproteins, other factors, etc.), additional experiments would need to be performed on the chimpanzee serum; however, the supply has been exhausted.

As HCV1 is intended for human therapeutic use, a relevant question is if some or all sera samples from HCV-infected humans are able to inhibit the neutralizing activity of HCV1 in HCVpp-based assays. Therefore, we conducted experiments on sera from multiple HCV-infected humans to assess interference with HCV1 neutralization of HCVpp (data not shown). The HCVpp neutralizing titers in these sera were so high that dilutions >1∶1000 needed to be employed to distinguish HCV1 activity from endogenous neutralizing activity against HCVpp, i.e. the human sera neutralized HCVpp potently which masked HCV1 neutralization. Mixed with human serum at dilutions >1∶1000, HCV1 neutralization of HCVpp was not effected (Babcock, unpublished data). However, chimpanzee serum inhibition was only significant at dilutions of <1∶500. As such, it is not clear if human sera contains factors that inhibit HCV1 neutralization at dilutions <1∶1000. It is possible that human sera contains inhibiting factors similar to those present in two of the chronically-infected chimpanzees; however, these factors could be diluted in the setting of a liver transplant with large blood and fluid losses and replacements or may only be relevant for the *in vitro* HCVpp system.

One limitation of this study was the sole use of HCVpp as a model *in vitro* system for understanding HCV1 neutralization capacity and the impact of chimpanzee serum factors on HCV1 neutralization. Cell culture-infectious HCV (HCVcc) has been developed which recapitulates all steps of the viral life cycle including entry [48]–[50]. HCVcc have been shown to be infectious *in vivo* [51] and as such are considered a more appropriate viral infection system than HCVpp. Numerous differences exist between HCVpp and HCVcc including, but not limited to, envelope glycoprotein density [52], glycan incorporation [53], and interaction with factors present in serum [52], [54]. It is unclear how these differences between HCVpp and HCVcc would impact the current results. Clearly, the results demonstrating E2 glycoprotein escape variants using HCVpp would be expected to repeat using the HCVcc system. However, HCV1 neutralization capacity of HCVpp was adversely affected by the presence of poorly-responding chimpanzee serum. If HCVcc interact with serum factors differently than HCVpp it is possible that we may observe a different impact of serum on HCV1 neutralization. HCVcc may be more resistant to HCV1 than HCVpp in the presence of chimpanzee serum but the possibility exists that HCVcc may be more sensitive to neutralization. In either case, further study using HCVcc to validate and confirm the current results obtained with HCVpp is warranted.

In the treatment study, using chronically-infected chimpanzees, we administered a dose of 40 mg/kg HCV1 rather than the 250 mg/kg used in the prevention study due to IACUC restrictions. It is possible that a higher dose of HCV1 may have reduced the viral load in all three chronically-infected chimpanzees rather than one of three by overcoming the neutralization inhibition imposed by the poorly-responding chimpanzee sera. 40 mg/kg was the highest dose allowed at this specific animal facility and it is not possible to test again with higher doses of HCV1.

We previously demonstrated that HCV1 was able to bind peptides and bacterially-expressed fusion proteins comprising E2 amino acids 412–423. The strong binding of HCV1 to both peptides and bacterially-expressed proteins suggests that glycosylation at positions 417 and 423 (two putative E2 glycosylation sites) is not required for binding [26]. Interestingly, HCV1 was able to bind soluble E2_660_ harboring N415K mutations even though neutralization capacity against HCVpp bearing this same alteration was lost [26]. Here we show that HCV1 is able to bind E2_660_–N415K protein produced from defined medium but not from serum-containing medium, the same media used for the production of HCVpp. These data illuminate our previous results and demonstrate that resistance mutations in the 412–423 epitope do indeed abrogate HCV1 binding to E2 and likely HCVpp produced in serum-containing media. It is unclear why E2_660_–N415K produced in defined medium can be bound by HCV1, but the altered migration of the protein in SDS-PAGE suggests E2_660_ is glycosylated differentially in defined medium. We speculate that complete and appropriate glycosylation at positions 417 and/or 423 impacts the ability of HCV1 to bind E2 envelope glycoprotein containing mutations at positions 415 and 417, but not wild-type E2. Of note, the N417S mutation eliminates the N-linked glycosylation site at position 417 but does introduce a potential O-linked glycosylation site. The binding of HCV1 to E2_660_–N417S produced in serum-containing media may be impacted by this possible O-linked glycosylation. Also, differential glycosylation of amino acids distant from amino acids 412–423 could possibly be in proximity to the HCV1 epitope when positions 415 and 417 are mutated disrupting HCV1 binding. Future studies of HCV1 (and possibly other epitope I antibodies) binding and epitope mapping should avoid the use of peptides and bacterially-produced proteins that lack glycosylation as this may lead to erroneous results.

HCV1 effectively prevents acute HCV infection and significantly reduces viral load to undetectable levels in a proportion of chronically-infected chimpanzees at doses ranging from 40 mg/kg to 250 mg/kg. A phase 1 trial in healthy adults has been completed and HCV1 proved to be safe and well tolerated. Studies are now in progress to examine the efficacy of HCV1 in the prevention of HCV infection of the donor liver in the liver transplant setting.

## Materials and Methods

### Ethics statement

All experiments using chimpanzees were performed in accordance with the *Guide for the Care and Use of Laboratory Animals* and were approved by the Institutional Animal Care and Use Committees at both the Southwest National Primate Research Center at the Texas Biomedical Research Institute (Animal Welfare Assurance Number A3082-01) and New Iberia Research Center (Animal Welfare Assurance Number A3029-01).

### Cells and cell culture

CHO-K1SV cells (Lonza) were grown in CD-CHO media (Invitrogen) or Dulbecco's modified eagle medium supplemented with 10% fetal bovine serum (DMEM/FBS). HEK-293T/17 cells (ATCC) were grown in DMEM/FBS supplemented with 100 IU penicillin/streptomycin. CHO-K1SV cells in CD-CHO were grown in Erlenmeyer flasks with constant orbital shaking. All cells were cultured at 37°C in air supplemented with 5% CO_2_.

### Antibody cloning, expression and purification

HCV1 heavy and light chain genes were cloned into a mammalian expression vector containing a glutamine synthetase (GS) gene as previously described [26]. CHO-K1SV cells were electroporated with the HCV1 expression vector and transfectants were selected using 50 µM methionine sulfoximine (MSX). Transformants were screened for antibody expression and the highest expressing clone (>500 mg/L) was selected for antibody production in bioreactors. Culture supernatant from bioreactors was clarified using centrifugation and filtration and antibody was purified using protein A followed by ion exchange chromatography. Antibody was formulated at 10 mg/ml in 20 mM citrate/150 mM NaCl/0.025% Tween-80 and was determined to be >98% antibody monomer lacking detectable endotoxin.

Antibody 96-2 is a human monoclonal antibody directed against HCV antigenic site II (amino acids 432–443) which was generated and characterized during the isolation of HCV1 [26]. 96-2 was purified from hybridoma culture supernatant which was incubated with protein A sepharose beads (GE Healthcare) for 2 hours at room temperature while rocking. Beads were removed by column filtration, washed with PBS and antibody eluted with 100 mM glycine pH 2.8. Eluate was dialyzed against PBS and concentrated using an Amicon YM-30 centriprep concentrator as described by the manufacturer. Purified antibody was filter sterilized and protein concentration determined by spectrophotometry.

Anti-(His)_6_ monoclonal antibody is a mouse monoclonal antibody that was developed in house and purified from hybridoma supernatants as described above.

### Prevention study in chimpanzees

Chimpanzees were maintained in accordance with the *Guide for the Care and Use of Laboratory Animals* at the Southwest National Primate Research Center at the Texas Biomedical Research Institute (Animal Welfare Assurance Number A3082-01). The protocol (#1070 PT0) was approved by the center's Institutional Animal Care and Use Committee. Healthy female chimpanzees were untreated or infused with 50 mg/kg or 250 mg/kg HCV1 antibody 30 minutes prior to inoculation with 32 chimp infectious doses (32 CID) of H77 genotype 1a HCV serum [31], [32]. Animals were followed for a total of 20 weeks following delivery of the HCV inoculum. Serum samples were obtained at varying time points to measure viral load using a TaqMan assay as well as HCV1 serum concentration using ELISA. In addition, clinical and safety laboratory testing was performed throughout the study (20 weeks).

### Treatment study in chimpanzees

Chimpanzees were housed and maintained according to the *Guide for the Care and Use of Laboratory Animals* at the New Iberia Research Center (Animal Welfare Assurance Number A3029-01). The protocol (#2011-8741-027) was approved by the center's Institutional Animal Care and Use Committee. IACUC approval restricted HCV1 dosing to 40 mg/kg in this study. Three female chimpanzees, chronically infected with H77 genotype 1a HCV, were infused intravenously with 40 mg/kg HCV1 over one hour. Chimpanzees were followed for 35 days with blood samples collected for safety labs (hematology and serum chemistries). Viral load (quantitative PCR – lower limit of quantification of 15 IU/ml) and circulating antibody concentration (ELISA) to E2 amino acids 412–423 were measured at multiple time points during the five week study period.

### Sequencing of HCV E1/E2 DNA from chimpanzee serum

The RNeasy kit (Qiagen) was employed following the manufacturer's instructions to isolate RNA from 100 µl to 1 ml of serum and purified RNA was stored as frozen aliquots. RT-PCR was performed on 3 µl of isolated RNA using the Superscript III One-Step RT-PCR System with Platinum Taq (Life Technologies) as described by the manufacturer. The forward oligonucleotide consisted of a 5′ overhang and HindIII site followed by a sequence corresponding to a region upstream of the E1 coding region (5′– GCT TAG CAA GCT TCG CCG ACC TCA TGG GGT ACA TAC CGC TCG −3′). The reverse oligonucleotide incorporated a 5′ overhang and XbaI site followed by a sequence complimentary to the terminal coding region of E2 (5′– CGC TTG CTC TAG ACG AGG TTC TCC AAA GCC GCC TCC GCT TGG −3′). The resulting RT-PCR product, consisting of 1891 base pairs, was digested with HindIII and XbaI and ligated to pcDNA3.1 (Invitrogen). DH5α *Escherichia coli* cells were transformed with the ligation reaction, plated to LB agar plates containing ampicillin, grown overnight at 37°C and colonies were assessed for E1/E2 PCR product incorporation. Positive clones (5–15 total) were sequenced by the Sanger method and analyzed using Vector NTI software.

### E1/E2 and E2_660_ cloning and mutagenesis

Prototypical genotype 1a E1/E2 envelope glycoprotein genes were amplified from an H77 expression plasmid (p90HCVconsensuslongpU) obtained through the AIDS Research and Reference Program, Division of AIDS, NIAID, NIH from Dr. Charles M. Rice via Apath, LLC. The PCR product contained 5′ HindIII and 3′ XbaI restriction sites and were cloned into pcDNA3.1 containing a 3′ (His)_6_ epitope tag. H77 soluble E2_660_ was cloned, expressed and purified as previously described [26].

To create expression constructs of E1/E2 isolated from chimpanzee serum, a forward primer (E1F1) containing a 5′ HindIII site, Kozak sequence and start codon followed by sequence complimentary to 5′ end of the E1 gene was designed (5′– GAT GAG CAA AGC TTG CCG CCA CCA TGG CCA CCG GCA ACC TGC CCG GCT G −3′). A reverse primer, E2R1, containing a 5′ XbaI site followed by sequence complimentary to the 3′ end of the E2 gene was also synthesized (5′– GCA TTC ACT CTA GAC GCC TCC GCC TGG GAG ATC AGC −3′). PCR with primers E1F1 and E2R1 using template consisting of cloned PCR products in pcDNA3.1 obtained during the sequencing of virus from chimpanzee serum was performed. Cloned E1/E2 was sequenced and confirmed.

Mutagenesis of the full-length E1/E2 and E2_660_ expression plasmids was performed using the Quick Change II Site-Directed Mutagenesis kit (Stratagene) following the manufacturer's instructions. Following mutagenesis the E1/E2 encoding region was sequenced to confirm the introduction of the desired mutation as well as integrity of the entire coding sequence.

### CD81 blocking assay

RNA was extracted from HEK-293T/17 cells using the RNeasy kit (Qiagen) as described by the manufacturer. RT-PCR was employed to amplify nucleotides 342 to 600 of the CD81 gene which encodes for the CD81 large extracellular loop (LEL). The amplicon was cloned into pGEX-6P (GE Healthcare) in frame with the N-terminal glutathione S-transferase (GST) and C-terminal myc epitope tag and expressed in BL-21 *E. coli*. Bacteria were lysed and the protein purified using glutathione sepharose chromatography. Purified CD81 LEL-GST was cleaved with PreScission protease to remove the GST tag and CD81 LEL was isolated by size-exclusion chromatohgraphy. Purified CD81 LEL (1 µg/ml) was coated on ELISA plates overnight at 4°C and subsequently washed. E2_660_ containing a C-terminal (His)_6_ tag (5 µg) was incubated with varying concentrations of HCV1 or an irrelevant human antibody for 1 hour at room temperature. Complexes were added to the CD81 LEL-coated ELISA plate and incubated for 1 hour at room temperature. E2_660_ binding was detected using an anti-(His)_6_ followed by goat anti-mouse IgG -alkaline phosphatase (AP) conjugate (1∶5000, Jackson Immunoresearch) and developed with p-nitrophenyl phosphate disodium salt (PNPP) at 1 mg/ml in 1 M diethanolamine. Absorbance (405 nm) was analyzed using Molecular Devices Emax plate reader with the Softmax software.

### ELISA

Chimpanzee serum and purified HCV1 was assessed for H77–E2_660_ (mutant or wild-type) binding using ELISA. 96-well microtiter plates were coated with 0.5–2 µg/ml of E2_660_ in PBS overnight at 4°C. 100 µl chimpanzee serum at varying dilutions was added to the wells and incubated at 22°C for 2 hours. Antibody binding was detected using an anti-human IgG-AP conjugate (1∶5000, Jackson Immunoresearch) followed by PNPP at 1 mg/ml in 1 M diethanolamine. Absorbance (405 nm) was analyzed using Molecular Devices Emax plate reader with the Softmax software.

### HCVpp neutralization assays

Pseudovirus was generated employing an HIV backbone that contained a mutation to prevent HIV envelope glycoprotein expression and a luciferase gene to direct luciferase expression in target cells (pNL4-3.Luc.R-E-) [55], obtained through the AIDS Research and Reference Program, Division of AIDS, NIAID, NIH from Dr. Nathaniel Landau. HCV E1/E2 glycoproteins were provided in *trans* by co-transfection of HEK-293T/17 cells with pcDNA-E1/E2-H77-1a (prototypical H77 sequence) or pcDNA-chimp-E1/E2 (E1/E2 from chimpanzee serum) with pNL4-3.Luc.R-E-. Supernatant containing virus particles was harvested 48 to 72 hrs post-transfection, concentrated using Centricon 70 concentrators, aliquoted and stored frozen at −80°C. Pseudovirus was pre-incubated with varying concentrations of antibody for 1 hr at room temperature before adding to Hep3B cells. After incubation for 72 hrs, infection was quantitated by luciferase detection with BrightGlo luciferase assay (Promega) and read in a Victor3 plate reader (Perkin Elmer) for light production.

For HCVpp assays to measure chimpanzee serum inhibition of HCV1 neutralization the above procedure was employed with minor modifications. HCVpp were pre-incubated with 100 nM HCV1 or irrelevant human antibody in the presence or absence of varying concentrations of chimpanzee serum for 1 hr at room temperature before adding to Hep3B cells. Light output was quantified for both irrelevant antibody and HCV1 at each concentration of chimpanzee serum and the ratio of light output of HCV1 to irrelevant mAb samples was determined.
